# The application of salvage surgery improves the quality of life and overall survival of extensively recurrent head and neck cancer after multiple operation plus radiotherapy

**DOI:** 10.3389/fonc.2022.1017630

**Published:** 2022-11-01

**Authors:** Lirui Zhang, Qiaoshi Xu, Huan Liu, Bo Li, Hao Wang, Chang Liu, Jinzhong Li, Bin Yang, Lizheng Qin, Zhengxue Han, Zhien Feng

**Affiliations:** Department of Oral and Maxillofacial-Head and Neck Oncology, Beijing Stomatological Hospital, Capital Medical University, Beijing, China

**Keywords:** salvage surgery, massive soft tissue flap, recurrent/metastatic head and neck cancer, quality of life, overall survival (OS)

## Abstract

**Objectives:**

The prognosis, choice of reconstruction and the quality of life (QOL) after salvage surgery (SS) for extensively locoregional recurrent/metastatic head and neck cancer (R/M HNC) is an important issue, but there are few reports at present.

**Materials and methods:**

We analyzed extensively locoregional R/M HNC patients from March 1, 2015, to December 31, 2021 who underwent SS with latissimus dorsi or pectoralis major musculocutaneous flaps. QOL were accessed using QLQ-H&N35 and UW-QOL questionnaire. Wilcoxon signed-rank test was used to compare difference between pre- and post-QOL and Kaplan-Meier curves were used in estimate overall survival (OS) and disease-free survival (DFS). The literature review summarized recent 10 years clinical trials of nonoperative treatment in R/M head and neck cancer.

**Results:**

1362 patients were identified and 25 patients were analyzed after screened. Median age at surgery was 59 years (range 43-77), 15/25(60%) were male and 22/25(88%) chose latissimus dorsi flap. Better mean pain score after applying massive soft tissue flaps revealed relief of severe pain(p<0.001) which strongly associated with improvement of QOL. The improved mean overall QOL score after surgery revealed a better QOL(p<0.001). As of June 1, 2022, 11/25 (44%) of the patients were alive. The 1-year, 2-year OS after SS was 58.4% and 37.2%, while the 1-year, 2-year DFS was 26.2% and 20.9%. The median OS of our study was better than nonoperative treatment of 11 included clinical trials.

**Conclusions:**

R/M HNC patients underwent SS can obtain survival benefit. The application of massive soft tissue flap in SS could significantly enhance the QOL for patients with extensively locoregional R/M HNC, especially by relieving severe pain.

## Introduction

Currently, surgery with adjuvant chemoradiotherapy is the most common treatment utilized for head and neck cancers (HNCs) (1–4). Nevertheless, recurrence is still common, with a rate of 25%-50% (5), especially for advanced stage cancer (Stage III or IV) (6). Extensively locoregional recurrent/metastatic R/M tumors in the head and neck region always lead to severe pain, appearance changes, swallowing dysfunction, chewing and despair, which profoundly impairs quality of life (QOL). Accordingly, R/M HNC involving key structural organs are a huge challenge in surgical treatment, both in therapy selection and implementation.

The term “salvage surgery” (SS) is currently defined as a final attempt to resect residual and recurrent tumors after definitive treatment, including surgical treatment (7). Surgery for patients with extensively recurrent head and neck tumors faces many problems, including the invasion of vital structures such as the skull base and the carotid artery which tightly related to the patient’ life safety and poor vascular conditions (8). Meanwhile, resection of a large tumor will form large area defect. Reconstruction with flaps has been applied to solve these issues in SS. Given the patients’ difficulties, the latissimus dorsi flap and pectoralis major musculocutaneous flap are two common choices due to their massive size and high success rate (9, 10).

Multiple studies on SS found good efficacy (11), with five-year overall survival (OS) and disease-free survival (DFS) rates of 42% and 47%, respectively, and it has been advocated as the last curative option for recurrent advanced head and neck cancer (12–14). Although the oncological outcomes of SS showed a substantial improvement, the evaluation of the quality of life before and after salvage surgery has been only poorly analyzed, and the significance of these flaps for the enhancement of QOL after SS is still unknown. The European Organization for Research and Treatment of Cancer (EORTC) questionnaire and University of Washington Quality of Life (UW-QOL) questionnaire are the two most frequently used questionnaires for globally measuring the QOL of patients with head and neck tumor (15).

Our research aimed to investigate whether SS can improve the overall survival time, and the application of latissimus dorsi and pectoralis major musculocutaneous flaps in SS is reliable and can improve the QOL of patients with extensively locoregional R/M HNC. In this study, we examined the QOL after SS for patients with R/M HNC using the validated instruments QLQ-H&N35 and UW-QOL.

## Materials and methods

### Study sample

The data used in this study originated from POROMS, a Prospective, Observational, Real-world Oral Malignant Tumors Study (clinicaltrials.gov identifier: NCT02395367). This database was established on January 1, 2015, based on the Department of Oral Maxillofacial Head and Neck Oncology, Beijing Stomatological Hospital. In this study, the initial study sample included all treated patients with oncologic malignancy from March 1, 2015, to December 31, 2021. From these patients, we selected eligible patients using the following criteria: (1) patients underwent resection surgery plus reconstruction with latissimus dorsi or pectoralis major myocutaneous flaps; (2) patients with a tumor that recurred more than once (including locoregional recurrence and a secondary primary tumor in the oral cavity, or distant oligometastases); (3) more than 2 cancer centers suggest quit or palliative care; (4) patients with a tumor invading vital structures (internal carotid artery, skull base, pterygoid plate, masticatory space, parapharyngeal space, trachea and suprasternal fossa, and orbit); (5) pre-operative and post-operative QOL questionnaires were integrally available with the instruments QLQ-H&N35 and UW-QOL; and (6) patients provided informed consent. Patients who were lost to follow-up or refused to participate were excluded from the study. This study was approved by the Beijing Stomatological Hospital ethics committee and conducted with the informed consent of the patients.

### Instruments

Pre- and post-operative QOL were measured using the QLQ-H&N35 (version 3.0) and UW-QOL (version 4.0). The EORTC Questionnaire has been well accepted and is widely used to evaluate the QOL of cancer patients (16–18). It contains a general questionnaire, QLQ-Core-30, and a specific module, QLQ-H&N35, which evaluates the common topics and topics specific to cancer patients, respectively. The QLQ-H&N35 is comprised of 7 multi-item scales assessing pain, problems with swallowing, senses (taste and smell), speech, social eating, social contact and sexuality and 11 single items assessing problems with teeth, opening the mouth, dry mouth, sticky saliva, coughing, feeling ill, and the use of analgesics, nutritional supplements, feeding tubes, weight gain and weight loss. The questions were scored on a four-point Likert scale (“not at all,” “a little,” “some,” “very much”), whereas the last five items were answered in the form of yes/no. The scale scores range from 0 to 100, with higher scores suggesting more severe symptomatology or problems (16).

Another questionnaire with the same application is the UW-QOL questionnaire, which was developed in 1993 by Hassan and Weymuller (19). It consists of 12 single question domains, including physical function domains with chewing, speech, swallowing, taste, saliva, and appearance, and social function domains with anxiety, mood, pain, activity, recreation, and shoulder function (20). In addition, it also has three global questions, one about how patients feel compared to the month before they developed cancer, one about their health-related QOL during the last 7 days and one about their overall QOL during the last 7 days. For the UW-QOL, each domain has 3-6 options scored evenly from 0 to 100, with higher scores implying better QOL.

### Data collection and integrity

Patient demographic characteristics (age, sex), history of treatment (whether they underwent radiotherapy, chemotherapy and other treatments), recurrent tumor characteristics (pathologic type, invasion of important structures), SS details (selection of flaps, the results of resection margins) and follow-up data (adjuvant therapy, post-operative complications, DFS and OS) were extracted from the mentioned database.

All patients registered in the database completed the pre-operative QOL questionnaires by themselves. Post-operative QOL questionnaires were completed 90 days ±7 days after surgery. We encouraged the patients to return to the outpatient clinic to complete the questionnaires to ensure the integrity and authenticity of the data, and telephone follow-up was only used for patients with poor physical condition who could not return to the hospital or died.

### Literature review

A search of human randomized controlled trial (RCT) in English language was completed in PubMed database from 2012 to 2022. The search queries contain MeSH terms of “chemotherapy” OR “molecular targeted therapy” OR “palliative care” OR “immunotherapy” AND “head and neck neoplasms” AND “recurrence” AND “phase 3 or phase III”. The retrieved articles will be further screened according to the exclusion criteria: (1) Nasopharyngeal cancer (21); (2) Repeated analysis of the same clinical trial sample; (3) Without complete description of survival time. The treatment plan and survival date of the finally included trials were extracted.

### Statistical analyses

Descriptive analysis was used to identify the sample characteristics. The Wilcoxon signed-rank test, a nonparametric test for two paired samples, was used to detect differences between pre- and post-QOL. Significance was established as p<0.05. OS and DFS were calculated by Kaplan–Meier analysis. The calculation starting point of OS and DFS was the time completing SS. The end point of OS was all-cause death or the last follow-up, while that of DFS was first recurrence, metastasis, or death. The OS time of the patients in this study was compared with the treatment of nonoperative therapy in the literature review, and the difference was displayed with a forest diagram. All statistical analyses were carried out using the statistical software program SPSS version 26.0.

## Results

### Sample characteristics

**Figure 1:**
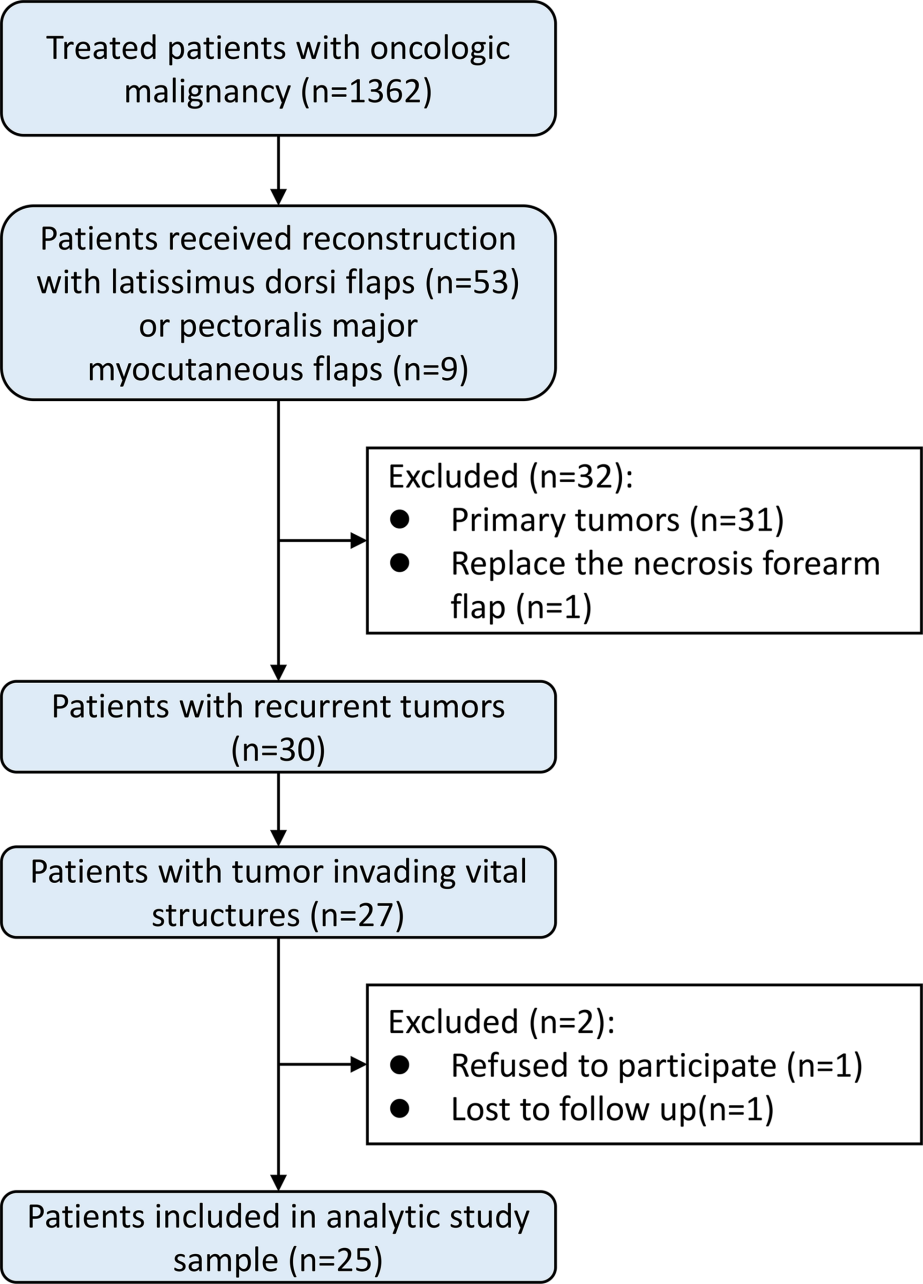
Flow chart of identification of eligible patients.

From January 1, 2015, to December 31, 2021, 1362 patients were enrolled in our program; 53 patients used a latissimus dorsi flap, and 9 patients used a pectoralis major musculocutaneous flap. Among these patients, 32 patients were excluded due to treatment of the primary tumor or replacement of a previous flap. Finally, 25 patients met the inclusion criteria and were eligible for the study (**Figure 1**). The median age was 59 years (range 43-77) at the time of surgery, and the proportion of men (60%) was slightly higher than that of women (40%). The predominant pathologic type was carcinoma (84%). The patient characteristics are shown in **Table 1**.

**Table 1:** Demographic and clinical characteristics.

Characteristics	N = 25
Age-year	
Median	59
Range	43-77
Gender-n (%)	
Male	15(60)
Female	10(40)
Previous surgery methods-n (%)	
Resection of primary tumor	8(32)
Resection of primary tumor + flap reconstruction	6(24)
Resection of primary tumor + neck dissection	7(28)
Resection of primary tumor +flap reconstruction + neck dissection	4(16)
History of radiotherapy-n (%)	
With	8(32)
Without	17(68)
History of chemotherapy-n (%)	
With	4(16)
Without	21(84)
History of other treatments-n (%)	
With	2(8)
Without	23(92)
Pathologic type-n (%)	
Carcinoma	21(84)
Sarcoma	3(12)
Chordoma	1(4)
Invasion of vital structures- n (%) ^a^	
Mastication muscles space and parapharyngeal space	24(96)
Skull base	8(32)
Internal carotid artery	2(8)
Pterygoid process	7(28)
Orbit	2(8)
Selection of flaps-n (%)	
Latissimus dorsi flap	22(88)
Pectoralis major musculocutaneous flap	3(12)
Results of salvage surgery-n (%)	
Negative margin	16(64)
Positive margin	6(24)
Positive margin → negative margin	3(12)
Adjuvant therapy-n (%)	
None	10(40)
Radiotherapy	7(28)
Chemotherapy	6(24)
Radiotherapy + Chemotherapy	2(8)
Complications-n (%)	
With	4(16)
Maxillofacial edema	2
Flap crisis	2
Bone exposure	1
Pharyngo-cutaneous fistula	1
Chronic pain	1
Without	21(84)

a There were 12 patients presented invasion of only one structure, 8 patients got two invaded structures and 5 patients got three.

### Salvage surgery

In terms of the history of prior treatments, 8(32%) patients received resection of the primary tumor alone. 6 (24%) patients underwent resection with reconstruction, while with neck dissection were 7(28%). The remaining 4(16%) patients underwent resection with both neck dissection and reconstruction. The number of patients who received radiotherapy, chemotherapy or other treatments was 8(32%), 4(16%), and 2 (8%), respectively.

The mastication muscle space and parapharyngeal space were the most frequently involved structures in 24 (96%) patients, while invasion of the skull base involved the internal carotid artery, pterygoid process, and orbit in 8(32%), 2(8%), 7(28%), and 2(8%) patients, respectively. Twelve patients presented with invasion of only one structure, eight patients had two invaded structures, and five patients had three invaded structures. In view of organ preservation and the need to avoid fatal complications, approximately 29% of patients could not obtain a negative margin.

Based on the follow-up notes, 7(28%) and 6(24%) patients underwent concomitant radiotherapy and chemotherapy, respectively; 2(8%) patients received both, and 10(40%) did not receive any adjuvant therapy. The incidence of complications was low, with only 4(16%) patients manifesting maxillofacial edema, flap crisis, pain, bone exposure and/or pharyngo-cutaneous fistula (**Table 1**).

### Restoration and reconstruction

**Figure 2:**
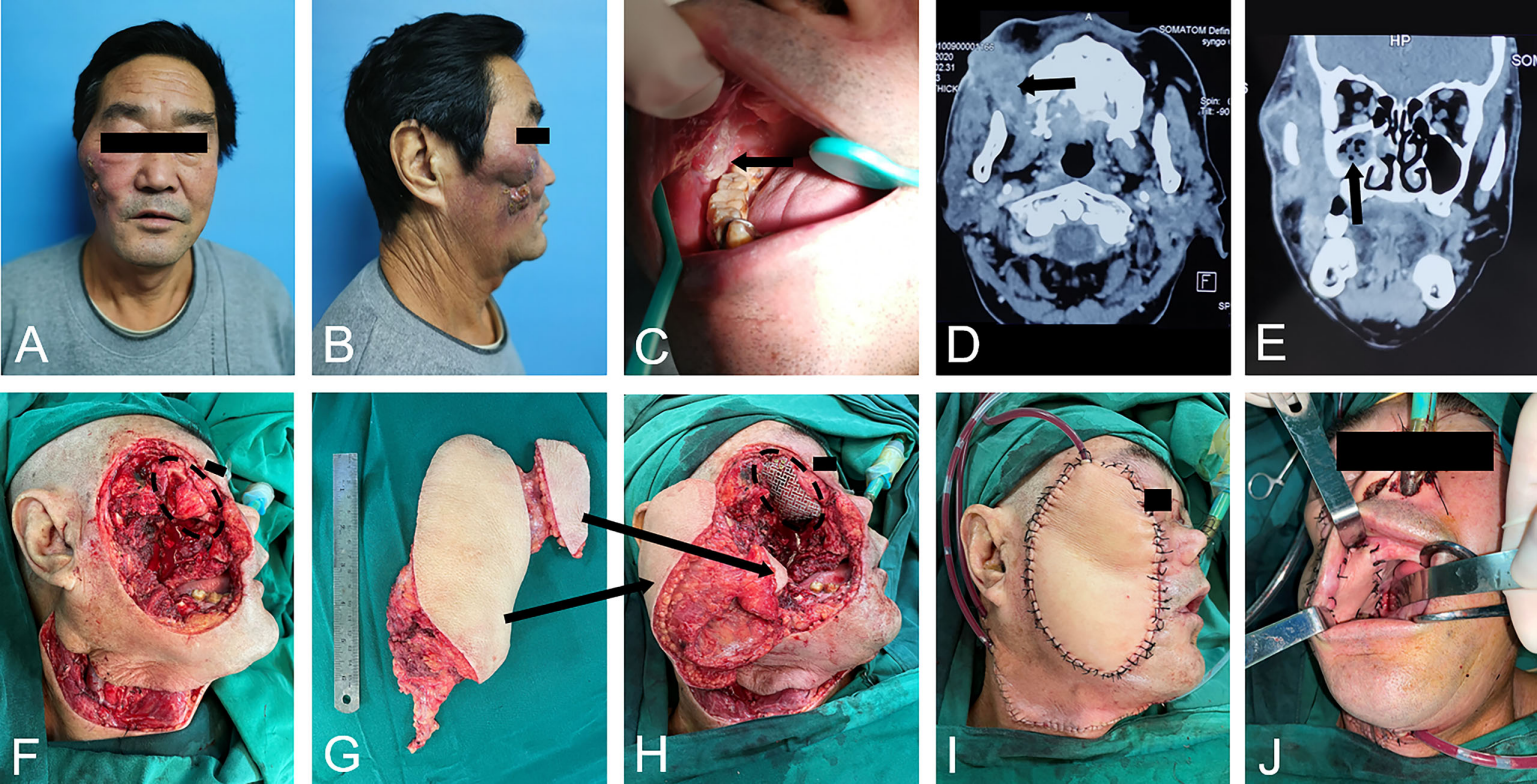
A typical salvage surgery case. **(A-C)**. primary tumor outside and inside the mouth; **(D, E)**. radiographic images presented tumor encroached maxillary and mandibular bone, maxillary sinus and orbital floor; **(F)**. excision of tumor led to orbit exposure; **(G, H)**. reconstruction with double-skin paddle free latissimus dorsi flaps and Titanium mesh implantation of right orbit; **(I, J)**. immediate postoperative images.

To protect the vital structures and improve the QOL, a latissimus dorsi flap and pectoralis major musculocutaneous flap were used for reconstruction. Among 25 patients, 22 patients chose the latissimus dorsi flap (88%), and 3 patients chose the pectoralis major musculocutaneous flap (12%). To show the restoration and reconstruction more intuitively, the operation process of a typical salvage surgical case is shown in **Figure 2**.

This is a case of a large recurrent head and neck squamous cell carcinoma (HNSCC) of the maxillofacial region in which the lesions involved the oral cavity, skin of the zygomatic face, outer orbital, sinuses and deep facial area (**Figure 2A-C**). Radiographic images presented tumor-encroached maxillary and mandibular bone, maxillary sinus and orbital floor (**Figure 2D, E**). The patient underwent extensive resection, including the maxilla, mandibular ramus, oral buccal mucosa and gingiva, zygomatic facial skin and lateral orbital bone wall (**Figure 2F**). Then, the large defect was reconstructed with double-skin paddle-free latissimus dorsi flaps and titanium mesh implantation of the right orbit after all margins were negative (**Figure 2G-J**). This case obtained a primary cure and received a satisfactory quality of life, and the tumor had not recurred at 1 year.

### Quality of life measured by UW-QOL

**Figure 3:**
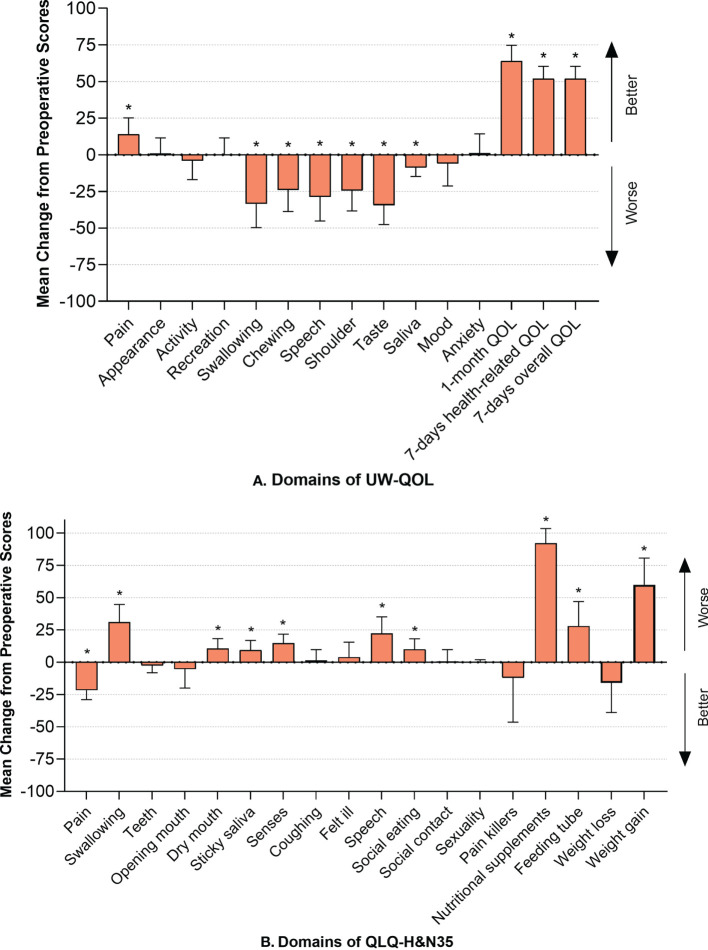
**(A)** Mean change from preoperative scores in UW-QOL and I bars indicate 95% confidence intervals. Asterisks represent significant difference between pre- and post- scores using Wilcoxon Signed Rank Test. Significance was established as p<0.05; **(B)** Mean change from preoperative scores in H&N35 and I bars indicate 95% confidence intervals. Asterisks represent significant difference between pre- and post- scores using Wilcoxon Signed Rank Test. Significance was established as p < 0.05.

The UW-QOL scores before and after SS are shown in **Table 2**. The mean overall QOL score prior to SS was 29.00 (95% CI, 24.12-33.88), while that after SS was 81.00 (95% CI, 72.96-89.04). This revealed significant differences (p<0.001) with a better QOL after SS. Among the 12 functional domains, the lowest pre-operative scores were found for pain (58.00), and the lowest post-operative scores were found for chewing (34.00). In comparative analyses of the UW-QOL scores between pre- and post- operative surgery, significant differences were found in 7 functional domains: pain (p=0.018), swallowing (p=0.001), chewing (p=0.005), speech (p=0.004), shoulder function (p=0.002), taste (p<0.001), and saliva (p=0.011). Among the former items, the domain “pain” achieved better scores, which implied relief after SS; however, worse scores were found in others, hinting at diminished functions (**Figure 3A**).

**Table 2:** Comparison of quality of life between preoperative and postoperative with UW-QOL.

UW-QOL	Preoperative	Postoperative	Difference-value*	P- value
	Mean (95%CI)	Mean (95%CI)	Mean (95%CI)	
**Functional domains**				
Pain	58.00 (53.09-62.91)	72.00 (63.41-80.59)	14.00 (2.82-25.17)	0.018
Appearance	67.00 (59.28-74.72)	68.00 (62.41-73.59)	1.00 (-9.52-11.52)	0.868
Activity	75.00 (68.34-81.66)	71.00 (58.83-83.17)	-4.00 (-16.86-8.86)	0.624
Recreation	76.00 (69.03-82.97)	76.00 (66.36-85.64)	0.00 (-11.54-11.54)	0.913
Swallowing	74.40 (64.42-84.38)	40.80 (29.38-52.22)	-33.60 (-49.71- -17.49)	0.001
Chewing	58.00 (48.25-67.75)	34.00(21.06-46.94)	-24.00 (-38.74- -9.26)	0.005
Speech	72.80 (60.85-84.75)	44.20 (32.32-56.08)	-28.6 (-45.06- -12.14)	0.004
Shoulder	100.00(100.00-101.03)	75.60 (61.70-89.50)	-24.40 (-38.30- -10.50)	0.002
Taste	97.60 (94.13-100.00)	63.20 (50.00-76.40)	-34.40 (-47.62- -21.18)	<0.001
Saliva	83.20 (76.93-89.47)	74.40 (68.09-80.71)	-8.80 (-14.80- -2.80)	0.011
Mood	72.00 (64.51-79.49)	66.00(53.76-78.24)	-6.00 (-21.27-9.27)	0.395
Anxiety	71.60 (62.79-80.41)	73.00 (62.38-83.62)	1.40 (-11.78-14.58)	0.848
**Composite domains**				
Compared to the month before you developed cancer	19.00 (14.50-23.50)	83.00 (73.71-92.29)	64.00 (53.23-74.77)	<0.001
Health-related quality of life during the past 7 days	29.00 (24.12-33.88)	81.00 (72.96-89.04)	52.00 (43.61-60.38)	<0.001
Overall quality of life during the past 7 days	29.00 (24.12-33.88)	81.00 (72.96-89.04)	52.00 (43.61-60.38)	<0.001

*Postoperative value minus preoperative value.

### Quality of life measured by the QLQ-H&N35

The results of the QLQ-H&N35 were analogous to those of the UW-QOL and are listed in **Table 3**. Pain (p<0.001) was significantly relieved, while dysfunction was found concerning swallowing (p=0.001), speech (p=0.003), social eating (p=0.018), and senses (p=0.001). Symptoms of dry mouth and sticky saliva were more severe, with P values of 0.017 and 0.023, respectively, compared to prior to surgery. A higher frequency of the use of nutritional supplements (p<0.001) and feeding tubes (p=0.008) was also found, accompanied by an increase in weight (p<0.001) (**Figure 3B**).

**Table 3:** Comparison of quality of life between preoperative and postoperative with EORTC QLQ-H&N35.

QLQ-H&N35	Preoperative	Postoperative	Difference-value*	P-value
	Mean (95%CI)	Mean (95%CI)	Mean (95%CI)	
**Multi-items scales**				
Pain	29.33 (22.89-35.78)	7.67 (5.25-10.08)	-21.67 (-28.90- -14.44)	<0.001
Swallowing	11.00 (5.77-16.22)	42.00 (29.94-54.06)	31.00 (17.29-44.71)	0.001
Senses	2.00 (-1.03-5.03)	16.67 (10.08-23.25)	14.67 (7.70-21.64)	0.001
Speech	15.56 (8.68-22.44)	37.78 (28.14-47.42)	22.22 (9.32-35.13)	0.003
Social eating	20.33 (13.87-26.70)	30.33 (24.22-36.44)	10.00 (1.81-18.19)	0.018
Social contact	13.93 (9.06-18.81)	14.67 (8.46-20.87)	0.73 (-8.25-9.72)	0.725
Sexuality	2.00 (-1.03-5.03)	2.00 (-1.03-5.03)	0.00 (-1.99-1.99)	1.000
**Single-item question**				
Teeth	6.67 (-0.21-13.55)	4.00 (-2.05-10.05)	-2.67 (-8.17-2.84)	0.317
Opening mouth	38.67 (25.09-52.24)	33.33 (29.36-37.31)	-5.33 (-20.02-9.36)	0.423
Dry mouth	22.67 (16.11-29.22)	33.33 (26.45-40.21)	10.67 (3.01-18.33)	0.017
Sticky saliva	20.00 (13.12-26.88)	29.33 (20.17-38.49)	9.33 (1.88-16.79)	0.023
Coughing	2.67 (-2.83-8.17)	4.00 (-2.05-10.05)	1.33 (-7.07-9.74)	0.785
Felt ill	33.33 (26.95-69.05)	37.33 (27.35-47.32)	4.00 (-7.46-15.46)	0.477
Pain killers	48.00 (15.78-56.22)	36.00 (15.78-56.22)	-12.00 (-46.37-22.37)	0.467
Nutritional supplements	8.00 (-3.43-19.43)	100.00 (100.00-100.00)	92.00 (80.57-103.43)	<0.001
Feeding tube	0.00 (0.00-0.00)	28.00 (9.08-46.92)	28.00 (9.08-46.92)	0.008
Weight loss	36.00 (15.78-56.22)	20.00 (3.15-36.85)	-16.00 (-38.86-6.86)	0.157
Weight gain	0.00 (0.00-0.00)	60.00 (39.36-80.64)	60.00 (39.36-80.64)	<0.001

*Postoperative value minus preoperative value.

### Survival outcomes

**Figure 4:**
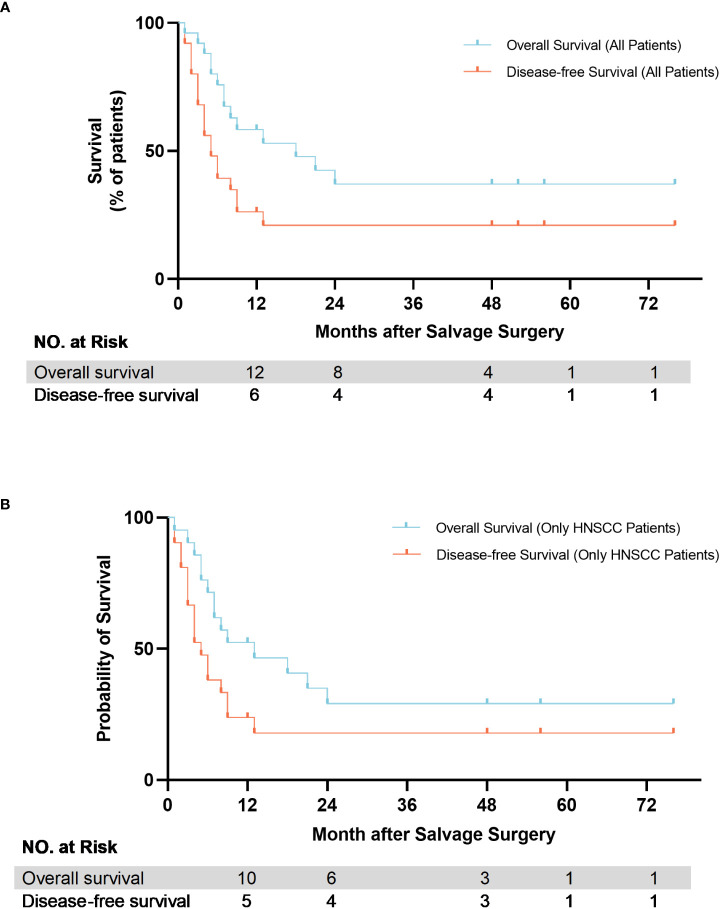
Kaplan-Meier estimate of overall survival (OS) and disease-free survival (DFS) of patients after salvage surgery. **(A)** All patients; **(B)** HNSCC patients only.

As of June 1, 2022, 11/25 (44%) of the patients in our study were still alive. The 1-year, 2-year OS after SS was 58.4% and 37.2%, respectively, while the DFS was 26.2% and 20.9%, respectively. The median OS of all 25 patients was 18.00 months (95%CI, 2.17-33.83), and the median DFS was 5.00 months (95%CI, 2.66-7.34). In pool of 21 HNSCC patients, the 1-year, 2-year OS after SS was 52.4% and 29.1%, respectively, while the DFS was 23.8% and 17.9%, respectively. The median OS was 13.00 months (95%CI, 0.00-26.30), and the median DFS was 5.00 months (95%CI, 2.76-7.24). The Kaplan–Meier curve showed the OS and DFS of all patients and HNSCC patients (**Figure 4**).

### Literature review

**Figure 5:**
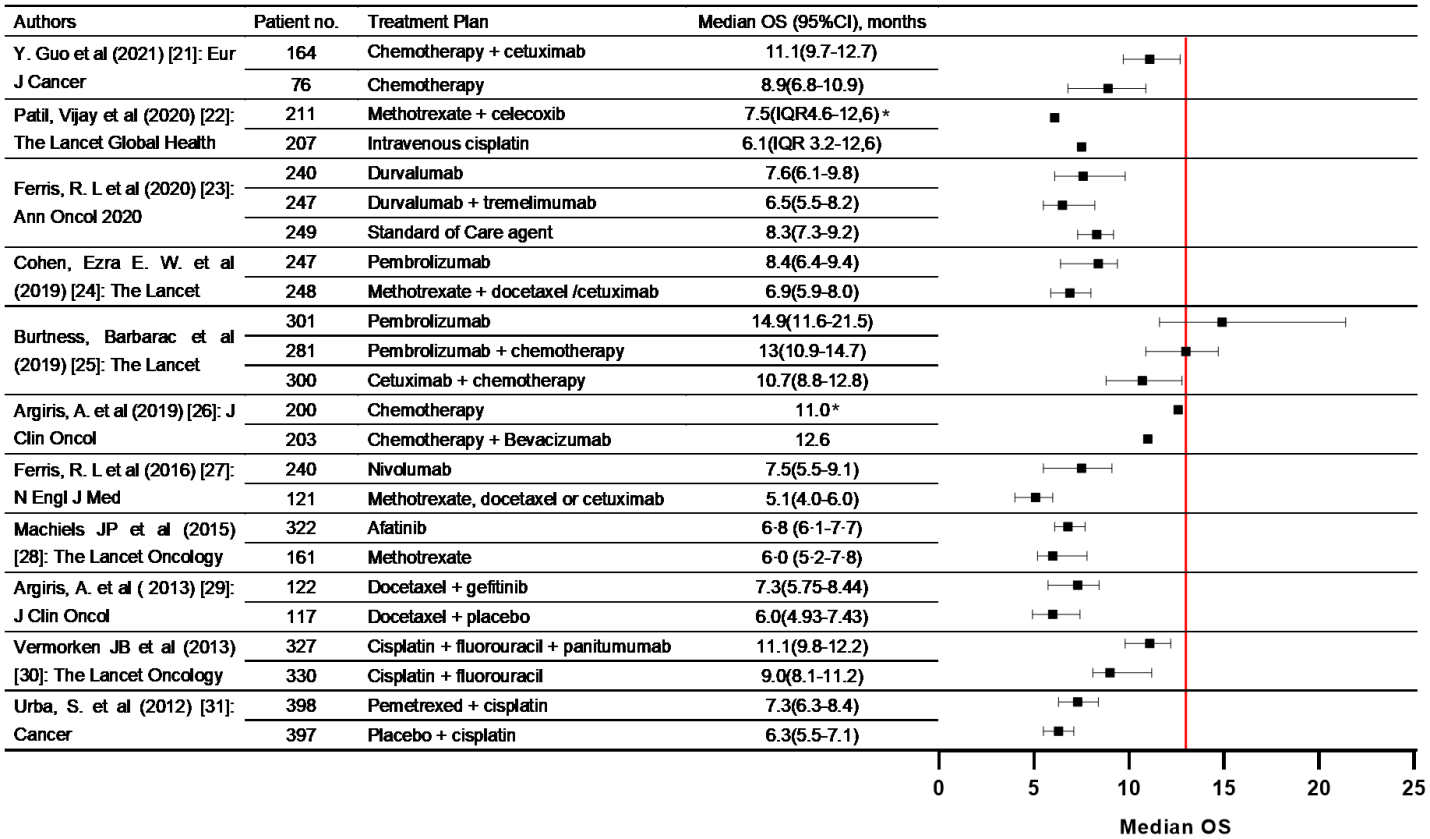
Summary of phase III clinical trials included in the literature review that evaluating nonoperative treatment in R/M HNSCC patients. The forest plots were built comparing median OS between our study and included trials. (* 95%CI was not presented in the original survival data.).

A total of 11 RCTs were included in our review (22–32). 5509 pathologically confirmed R/M HNSCC patients were included in trials and underwent single chemotherapy, molecular targeted therapy, immunotherapy or combination therapy. The reported date of median OS ranged from 5.1 months to 14.9 months. In our study, the median OS of all 21 patients only affected by squamous cell carcinoma was 13.00 months (95%CI, 0.00-26.30). Compared to these nonoperative treatment in R/M HNSCC, median OS of our study is apparently better. The forest plot showed the median OS with 95%CI of all the former trials (**Figure 5**).

## Discussion

SS is defined as an attempt to resect residual and recurrent HNCs. In our study, all 25 patients had undergone radical surgery 1-4 times, extensive relapse still occurred, and the tumor had invaded the internal carotid artery, skull base, pterygoid plate, masticatory space, parapharyngeal space or orbit which are generally considered unresectable (33). Under such circumstances, the patient has almost lost all courage to survive, and SS is the last attempt to defeat the cancer.

In this research, we applicate latissimus dorsi and pectoralis major musculocutaneous flaps for restoration and reconstruction after SS for patients with R/M HNC The head and neck region has many important structures and many functions. Based on this, surgery for head and neck tumors has a prominent impact on their QOL, and reconstruction has great meaning. For patients with extensive R/M HNC, the significance of repair and reconstruction lies in: 1. Coverage of important anatomical structures (e.g. the skull base and carotid artery), 2. Filling the dead cavity after resection of a large tumor, 3. Restoration of maxillofacial function, 4. Repairing the patient’s appearance. However, due to previous treatment experience and the characteristics of recurrent tumors, patients also face many difficulties, including the following: 1. Lack of anastomotic vessels and disordered anatomical structure in the recipient area, 2. Fewer flaps to choose from, 3. A large amount of tissue is necessary, 4. A high success rate is essential.

Considering these problems, the latissimus dorsi flap and pectoralis major musculocutaneous flap are the best choices. As common flaps, the tissue masses of these two flaps are larger than those of forearm flaps and anterolateral thigh flaps. At the same time, as nonfirst-line flaps, the latissimus dorsi flap and pectoralis major musculocutaneous flap can still be used when other common flaps have already been used in the initial operation. Moreover, multi-island flaps can often be modified to repair complex defects. In this study, we demonstrated the reliability of both flaps as a salvage surgical application. More importantly, we found that the use of both can significantly improve the QOL after SS.

As demonstrated by the UW-QOL questionnaires, the scores of the functional domains declined after SS. We considered it’ s inevitable as the tumor involves many functional structures. Dysphagia occurs frequently after treatment for HNCs because of the gross destruction of organs vital for swallowing (e.g., tongue, larynx, mouth floor and pharyngeal wall) (34, 35). The same consequence was found in our study: swallowing decreased significantly with lower scores, in which 18 of 25 patients suffered partial or total dysphagia, and all of them had one or all of these structures involved. In addition, it was found that problems with the shoulder were significantly worsened. Among the patients with decreased scores, two underwent accessory nerve snipping. We hypothesized that the performance of neck dissection is one possible reason for the excision or destruction of the accessory nerve. Dry mouth is a significant manifestation of salivary gland dysfunction, confirmed as a side effect after radiotherapy (36), and reduced salivary production or sticky saliva is associated with a decline in QOL (37). In our study, significant worsening of sticky saliva and dry mouth were found after surgery. Since our postoperative scale was collected in a short time after operation, the patients lacked perfect functional recovery training. Therefore, long-time post-operative functional recovery training might improve the situation which needs attention during further follow-up.

Surprisingly, we found the composite domains improved after SS and attributed this positive trend to the relief of severe pain, which is one of the most common symptoms of head and neck cancers and has a noticeable impact on QOL. Studies on understanding the priorities of patients with HNCs showed the most concern for cure, survival and avoiding pain (38), which hinted at a strong link between relief of pain and improvement of QOL. In the premise of balance with function, SS can remove the invading tumor and relieve the compression of nerves, which significantly contributes to the relief of severe pain. In addition, the removal of extensively invaded tumors is crucial to enhance the patients’ confidence in treatment modalities and their life expectancy.

Several previous studies indicated that the high rates of complications after SS in HNCs ranged from 23% to 67%, and pharyngocutaneous fistula was the most common complication (13, 39). Complications after SS in our study, however, were rare, affecting only 4 patients, and manifested as maxillofacial edema, flap crisis, chronic pain, bone exposure and/or pharyngocutaneous fistula. We considered this inconsistent result to be related to the small sample size and recall bias, with some post-operative follow-up information being provided by family members of the patient, and grief after their death might have influenced their reminiscence.

We tentatively estimated survival time and found that patients who underwent SS achieved 1-year OS and DFS rates of 58.4% and 26.2%, respectively, and 2 year survival rates were 37.2% and 20%, which is lower than previous results. Elbers reported the OS and DFS at 2-year of were 55% and 53%, respectively (12), and Hamoir reported a 2-year OS of 59% among patients who underwent SS (39). Of note, different previous studies have different emphases, such as specific subsites, specific salvage modalities or specific primary treatments (40–42). Thus, directly comparing their results can be one-sided and potentially misleading. In our study, all patients underwent primary treatment but still had extensive recurrence. Meanwhile, 6 patients (24%) still had positive margins after SS. Some studies also have reported that invasion of vital structures, especially pterygoid plates and the skull base, poses great challenges to achieve adequate resection and leads to poor survival outcomes (43, 44). However, compared to the literature review of nonoperative treatment in R/M HNSCC, median OS of our study is apparently better.

Limitations do exist in this study. The limited sample size is worrisome but it is due to the rarity of patients. Because of the terrible physical condition of some patients who could not return to the hospital, some post-operative follow-up data were obtained *via* telephone, which undoubtedly increased the inaccuracy of the data. Likewise, the criterion for responses to the questions differed for each patient due to their subjective character. Nevertheless, this study has provided some important results and suggestions for the future selection of SS.

## Conclusions

R/M HNC patients underwent SS can obtain survival benefit. The application of latissimus dorsi flap and pectoralis major musculocutaneous flap in SS for R/M HNC is feasible. It could significantly improve the patients’ quality of life after SS, especially by relieving severe pain. In the future, post-operative functional recovery training also needs attention during follow-up.

## Data availability statement

The raw data supporting the conclusions of this article will be made available by the authors, without undue reservation.

## Ethics statement

The studies involving human participants were reviewed and approved by Beijing Stomatological Hospital ethics committee. The patients/participants provided their written informed consent to participate in this study. Written informed consent was obtained from the individual(s) for the publication of any potentially identifiable images or data included in this article.

## Author contributions

Contribution Author(s) Study concepts: LZ, QX. Study design: ZF, ZH, LZ, QX. Date acquisition: LZ, QX, HW, CL, BY, L Q. Quality control of data and algorithms: LZ, QX, BL, JL. Data analysis and interpretation: LZ, QX, ZF. Statistical analysis: LZ, QX, HL. Manuscript preparation: All of the authors. Manuscript editing: All of the authors. Manuscript review: All of the authors. All authors contributed to the article and approved the submitted version.

## Funding

This article is supported by the Capital’s Funds for Health Improvement and Research (CFH2020-2-2143); the National Natural Science Foundation of China (82072984); the Project of Beijing Municipal Education Commission (KM202110025008); innovation Research Team Project of Beijing Stomatological Hospital, Capital Medical University (NO. CXTD202204) and Beijing Stomatological Hospital, Capital Medical University Young Scientist Program (NO. YSP202111).

## Conflict of interest

The authors declare that the research was conducted in the absence of any commercial or financial relationships that could be construed as a potential conflict of interest.

## Publisher’s note

All claims expressed in this article are solely those of the authors and do not necessarily represent those of their affiliated organizations, or those of the publisher, the editors and the reviewers. Any product that may be evaluated in this article, or claim that may be made by its manufacturer, is not guaranteed or endorsed by the publisher.
